# Submicron-Sized Nanocomposite Magnetic-Sensitive Carriers: Controllable Organ Distribution and Biological Effects

**DOI:** 10.3390/polym11061082

**Published:** 2019-06-25

**Authors:** Marina V. Novoselova, Sergey V. German, Olga A. Sindeeva, Oleg A. Kulikov, Olga V. Minaeva, Ekaterina P. Brodovskaya, Valentin P. Ageev, Mikhail N. Zharkov, Nikolay A. Pyataev, Gleb B. Sukhorukov, Dmitry A. Gorin

**Affiliations:** 1 Skoltech Center of Photonics & Quantum Materials, Skolkovo Institute of Science and Technology, Skolkovo Innovation Center, 3 Nobelya Street, Moscow 121205, Russia; s.german@skoltech.ru (S.V.G.); d.gorin@skoltech.ru (D.A.G.); 2 Remote Controlled Theranostic Systems Lab, Saratov State University, 83 Astrakhanskaya Street, Saratov 410012, Russia; o.a.sindeeva@gmail.com; 3 Surface Engineering Labs, Innovative Engineering Technologies Institute, Peoples’ Friendship University of Russia (RUDN University), 6 Miklukho-Maklaya St., Moscow 117198, Russia; g.sukhorukov@qmul.ac.uk; 4 Department of Biotechnology, Bioengineering and Biochemistry, Ogarev Mordovia State University, 68 Bolshevistskaya Street, Saransk 430005, Republic of Mordovia, Russia; oleg-kulikov-84@mail.ru (O.A.K.); polinanew@mail.ru (O.V.M.); kitten-777@mail.ru (E.P.B.); valeageev@yandex.ru (V.P.A.); mikhail.zharkov.92@mail.ru (M.N.Z.); pyataevna@mail.ru (N.A.P.); 5 School of Engineering and Materials Science, Queen Mary University of London, Mile End Road, London E1 4NS, UK

**Keywords:** submicron-sized particles, bovine serum albumin, tannic acid, carriers, magnetic field gradient, magnetite (MNPs), biodistribution, fluorescent imaging

## Abstract

Although new drug delivery systems have been intensely developed in the past decade, no significant increase in the efficiency of drug delivery by nanostructure carriers has been achieved. The reasons are the lack of information about acute toxicity, the influence of the submicron size of the carrier and difficulties with the study of biodistribution in vivo. Here we propose, for the first time in vivo, new nanocomposite submicron carriers made of bovine serum albumin (BSA) and tannic acid (TA) and containing magnetite nanoparticles with sufficient content for navigation in a magnetic field gradient on mice. We examined the efficacy of these submicron carriers as a delivery vehicle in combination with magnetite nanoparticles which were systemically administered intravenously. In addition, the systemic toxicity of this carrier for intravenous administration was explicitly studied. The results showed that (BSA/TA) carriers in the given doses were hemocompatible and didn’t cause any adverse effect on the respiratory system, kidney or liver functions. A combination of gradient-magnetic-field controllable biodistribution of submicron carriers with fluorescence tomography/MRI imaging in vivo provides a new opportunity to improve drug delivery efficiency.

## 1. Introduction

In the past decade, the development of effective carriers of bioactive compounds for use in preventive and personalized medicine has received considerable research attention.

Ideally, a carrier should have the following characteristics: sufficient drug loading capacity, high potential for accumulation in the diseased tissue, controlled drug release kinetics, biological inertness and complete biodegradation [1].

A possible approach to making such composite carriers is sequential adsorption of polyelectrolytes (layer-by-layer, LbL) from solutions on the surface of template spherical particles of calcium carbonate (vaterite), with subsequent dissolution of the latter [2,3]. The size of these carriers determines the in vivo accumulation and translocation and the biological fate and toxicity of these delivery systems. The optimal size of a drug delivery carrier depends on the type of carrier administration and localization of the target in an organ. Recently, it was demonstrated that the size of particles affects their biodistribution in the lung [4]. The smallest, submicron-sized particles penetrate deeper than do micron-sized particles [4]. In addition, size can influence drug loading, drug release, and nanoparticle stability [5]. The effect of particle size is nonlinear and varies from organ to organ, underscoring the importance of tuning particle size to suit each distinct application in vivo [4]. Generally, a large particle is easily removed by the liver and spleen. Reducing the size of colloidal particle carriers enhances the stability of the carrier nanoparticles [6] and creates the chance of escape from the vascular system via cavities in the lining of blood vessels. Thus, the circulation time of small particles is higher than that of large particles in drug delivery systems [6]. Particle size also affects drug release [7]. Smaller particles have a larger surface area-to-volume ratio; therefore most of the drug associated with small particles would be at or near the particle surface, leading to faster drug release [8]. In contrast, larger particles have a large drug loading capacity per particle, which allows more drug to be encapsulated in each particle and slower release to occur [9]. Thus, control of particle size provides a means of tuning drug release rates [5]. From this standpoint, an undoubtedly important and promising task is to adapt and optimize the proposed approach to the effective adsorption of a preparation to submicron carriers. This would make it possible to overcome a number of problems related to the dimensional characteristics of composite microcarriers.

Controllable biodistribution (CB) of these drug delivery carriers offers the advantages of decreased drug toxicity and improved therapeutic efficacy because of the selective accumulation of the therapeutic agent within the diseased tissue. The magnetic targeted drug delivery system is a very attractive strategy for delivering colloidal magnetic nanoparticles to the area of interest. A potential benefit of this system is the use of localized magnetic field gradients to attract particles to a chosen site and the possibility to hold them there until the therapy is complete, and then to remove them. For the magnetic targeting system, drugs are incorporated with magnetic carriers and biodegradable polymers [10,11,12]. Suitable biodegradable polymers, such as polylactides [13], poly(e-caprolactone) [14], polyalkylcyanoacrylate [15], polyglycolides [13], poly(lactide-co-glycolides) [16], polyanhydrides [17] or polyorthoesters [17] are usually chosen to make drug-carrying particles [18]. The main drawback of all these carriers is the use of expensive and cytotoxic polycations [19]. Recently, we reported that LbL-assembled multilayered microcapsules can be prepared using a low-cost and a food grade ingredients as the tannic acid (TA) and the bovine serum albumin (BSA) exploiting the ability of tannins to precipitate proteins by hydrogen bonding and hydrophobic interactions [20,21]. The microcarriers demonstrate low cytotoxicity and are resistant toward treatment with trypsin but susceptible to α-chymotrypsin—two proteolytic enzymes differing in cleavage site specificity [22]. Proteins represent good raw materials since they have the advantages of synthetic polymers together with the advantages of absorbability and low toxicity of the end product degradation [23,24,25]. BSA could be substituted by human serum albumin to avoid a possible immunologic response [26]. Incorporation of TA into the shell has an additional benefit of providing the microcarriers with antioxidant properties by scavenging Fe^2+^ ions [22,23,27,28]. The BSA-TA shell is a promising alternative to the popular biodegradable pArg-Dex shell—a relatively expensive and, according to some data, a toxic system [20]. However, the in vivo biosafety of the BSA-TA carriers has not been previously described in the literature.

Thus, the aim of this work was to produce and investigate the efficacy and biosafety of magnetic submicron carriers based on BSA and TA as drug carriers. The carriers are made of biodegradable polymers BSA-TA with magnetic nanoparticles incorporated into core. The size of polymer carriers was placed in the following interval 400–600 nm. The use of a submicron carriers determines the relevance of the project, as it gives the opportunity to overcome a number of problems related to dimensional characteristics of the composite microcarriers associated with its movement in the bloodstream and the following biodistribution.

Thus, the aim of the work was to produce and investigate the efficacy and safety of magnetic submicron carriers based on BSA and TA as drug carriers. The carriers were made of biodegradable polymers (BSA-TA), with magnetic nanoparticles incorporated into the core and having a size of about 400–600 nm. The use of submicron carriers determines the relevance of the project, making it possible to overcome problems related to the dimensional characteristics of the composite microcarriers associated with their movement in the bloodstream.

## 2. Materials and Methods

### 2.1. Materials

Bovine serum albumin (BSA), tannic acid (TA), phosphate-buffered saline (PBS), sodium hydroxide (99.8%), iron(III) chloride hexahydrate (FeCl_3_·6H_2_O), iron(II) chloride tetrahydrate (FeCl_2_·4H_2_O), dimethylsulfoxide (DMSO), citric acid, ethylene diamine tetraacetic acid disodium salt (EDTA), hydrochloric acid, calcium chloride dehydrate, anhydrous sodium carbonate, sodium chloride, and sodium hydroxide were all purchased from Sigma–Aldrich (St. Louis, Missouri, USA). Dulbecco’s modified Eagle’s medium, fetal bovine serum, trypsin–EDTA, and DPBS were purchased from GIBCO (Barcelona, Spain). Alamar blue and 4′,6-diamidino-2-phenylindole dihydrochloride (DAPI) were purchased from Thermo Fisher Scientific (Waltham, Massachusetts, USA). Cyanine 7–N-hydroxysuccinimide ester (Cy7–NHS) was purchased from Lumiprobe (Hannover, Germany). Cy7-labelled BSA (BSA–Cy7) was prepared by a standard Lumiprobe protocol and was purified by dialysis. All chemicals were used without further purification. Deionized (DI) water (specific resistivity, higher than 18.2 MΩ cm) from a Milli-Q plus 185 water purification system Millipore (Burlington, Massachusetts, USA) was used to make all solutions. HeLa and fibroblast cells were used in the work.

### 2.2. Methods

#### 2.2.1. Synthesis of Magnetic Nanoparticles

Magnetic nanoparticles were obtained by chemical precipitation from di- and trivalent salts of iron in the presence of a base, as described by German at al. 28 The mixing of the reagents and the washing steps were carried out in a nitrogen atmosphere in a chemical reactor. The average size of the nanoparticles measured by dynamic light scattering was 11 ± 3 nm. The concentration of the magnetite colloid was 1.25 mg/mL.

#### 2.2.2. Preparation of Nanocomposite Microcarriers.

**Figure 1:**
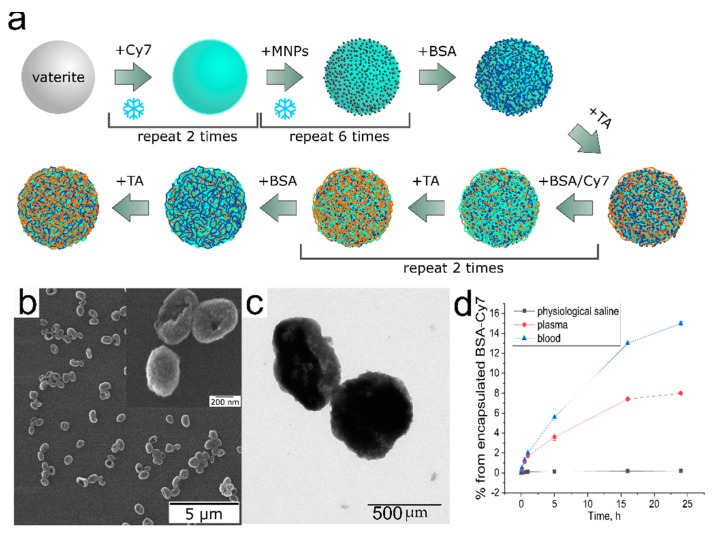
Scheme of the carrier synthesis and freezing induced loading (**a**), SEM (**b**), TEM (**c**) images, and the effect of various biological fluids on stability of MNPs (BSA-TA) (**d**).

LbL assembly was used to prepare microcarriers (Figure 1). Spherical porous CaCO_3_ particles with an average diameter of ~400–600 nm (see SEM images in Figure 1) were synthesized as described elsewhere 29. In brief, 0.4 mL of 0.5 M CaCl_2_ and 0.5 M Na_2_CO_3_ solutions were injected into 4 mL of glycerol under vigorous agitation. One hour later agitation was stopped, the particle suspension was centrifuged, and the sediment was washed two times with DI water. Porous vaterite submicron particles were loaded with MNPs and BSA–Cy7 by the freezing-induced loading (FIL) method (Figure 1a). Microcarriers were obtained by adsorption of 1 mL of BSA (concentration, 2 mg/mL in H_2_O), TA (concentration, 2 mg/mL in H_2_0), and BSA–Cy7 (concentration, 2 mg/mL in H_2_O) onto the spherical surfaces of the calcium carbonate cores. The cores were then extracted with EDTA (concentration, 0.2 M in water; pH 7.3). After each adsorption step, as well as after the dissolution of the calcium carbonate cores, the suspension of the microcarriers was centrifuged and washed two times with pure water. The carriers were prepared with the following two types of structures: the first is named MNPs (BSA-TA). It consists from two times freezing induced loaded by Cy7 labelled BSA and six times freezing induced loaded by magnetite nanoparticles MNPs to the shell with the following composition- (BSA-TA/(BSA-Cy7-TA)_2_/BSA-TA); the second was freezing induced loaded two times by Cy-7 labelled BSA into the shell with the same composition as the first one (BSA-TA).

The carrier concentration was measured by flow cytometry using Cytoflex (Beckman Coulter, Brea, California, USA).

Scanning electron microscopy (SEM) was done with a MIRA II LMU instrument (Tescan, Brno, Czech Republic). Samples were prepared by depositing a drop of a suspension of particles or carriers on a silicon wafer and allowing it to dry at room temperature. Before imaging, the samples were coated with an approximately 5-nm-thick gold film by using a Denton sputter coater. Images of composite and polymer microcarriers were taken at 30 kV.

Fluorescence spectra were measured with a Synergy H1 multi-mode reader (BioTek Instruments, Inc., Winooski, Vermont, USA). All measurements were made with disposable 96-well plates at 24 °C.

Fluorescence images of microcarriers were obtained with a Leica TCS SP8 X inverted confocal microscope (Leica Microsystems, Wetzlar, Germany; oil immersion objective, HC PL APO 100x/1.44).

Sample magnetization was measured by the induction method with an EZ11 vibration magnetometer (Microsense Inc., Lowell, Massachusetts, USA). Samples were placed in a special vial, dried in a vacuum oven for 4 h at 20 °C, and placed in the measuring cell of the magnetometer. Saturation magnetization (MS) was measured.

Investigation of carrier stability in biological fluids (0.9% NaCl, plasma, and blood).

The stability of the carriers was determined from the change in fluorescence intensity after exposure to biological fluids and salts. A 50-μL portion of a carrier suspension was diluted in 150 μL of 0.9% NaCl, blood, or plasma. Samples were incubated for 24 h at 37 °C with constant stirring. Fluorescence spectra were measured after 0 min, 5 min, 30 min, 1 h, 2 h, 5 h, 16 h, 24 h, and 40 h of incubation by using a Synergy H1 multi-mode reader (BioTek Instruments, Inc., Winooski, Vermont, USA). BSA/TA carriers without MNPs, BSA–Cy7, and Cy7 were used as the controls.

#### 2.2.3. In Vitro Biocompatibility

##### Cell Viability Assay

The alamar blue method was used to measure cell viability. Aliquots of the cell suspension (200 µl; concentration, 1 × 10^3^ cells/mL) were incubated in a 96-well microtiter plate for 24 h. Four wells without MNCs were used as the controls. After 24 h, MNCs were added to the wells with cells and were incubated for 24, 48 and 72 h. Then, the samples were treated with 10% Alamar blue (Trek Diagnostic Systems, Inc.) and their fluorescence was imaged after 24, 48, and 72 h with a Synergy H1 multimode reader (BioTek Instruments, Inc., Winooski, Vermont, USA) by using 530 nm for excitation and 590 nm for emission. Cell viability was expressed in percent of control (cells with no carriers added).

##### Interaction of Carriers with the Coagulation Cascade.

To evaluate changes in the coagulation properties of blood as a result of contact with carriers, we measured such coagulation indicators as prothrombin time (PT, an indicator of the external coagulation pathway) and activated partial thromboplastin time (APTT, an indicator of the internal coagulation pathway). These were measured with an Amelung KC4 coagulation analyzer (TRINITY Biotech, Ireland) in duplicate. Blood from four healthy donors was dispensed into tubes with sodium citrate as the anticoagulant and was incubated with particles (MNPs(BSA–TA) and BSA–TA). The scheme of the experiment was the same as described by Dobrovolskaia et al. [29]. A solution of 0.9% NaCl was used as the reference. After incubation, plasma was isolated by collecting the supernatant liquid and was subjected to clotting tests.

##### In Vivo Study

BALB/c male and female mice were used. All experiments complied with the relevant guidelines and regulations. The design of all experiments was approved by the ethical committee of Ogarev Mordovia State University, Russia.

##### MRI

MRI was conducted with a Philips Achieva 1.5 T high-field MRI scanner equipped with a phased array coil. The T2- and T1-weighted quick spin-echo protocols (turbo spin echo, TSE) were applied. Measurements were made with the following parameters: for theT1-weighted pulse sequence, the repetition time (TR) was 450 ms and the echo time (TE) was 15 ms; for theT2-weighted pulse sequence, the TR was 3000 ms and the TE was 47.7 ms.

MRI was conducted before injection of microcarriers (precontrast) and after 1 h and 2, 4, and 25 days. To this end, 200 μL of a suspension containing 1 × 10^9^ carriers was injected into the mouse tail vein. The left paw was fixed on the magnet, and the right one served as the control (no external magnetic field). The mice were fixed on the magnet for 1 h, after which MRI images were taken.

##### In Vivo Fluorescent Imaging

This was conducted with two groups of mice. The experimental group was treated with MNPs (BSA/TA) carriers. Before the experiment, animals were anesthetized by intramuscular injection of zoletil (50 mg/kg). The target place of carrier delivery was the left hind paw. For magnetic targeting, this paw was attached to a cylindrical Fe-B-Ne magnet (D = 50 mm, h = 30 mm, MS = 0.5 Tl), while the right hind paw was magnet-free. A 0.2-mL portion of a suspension of MNPs(BSA-TA) carriers in saline solution (0.9% NaCl) was administered intravenously into the tail vein. The control group was treated with solutions of BSA-TA carriers. The mice were imaged at 0, 0.05, 0.5, 1, 5, 24, and 48 h with an IVIS Lumina II imaging system (Xenogen Corp., PerkinElmer, Hopkinton, Massachusetts, USA) by using excitation/emission at 675/810–875 nm. Photons were quantified with LivingImage software (Xenogen Corp).

##### In Vivo Magnetite Biodistribution Study

Magnetite biodistribution was evaluated by magnetometry and by a histological method in three mice. The method of administration, the dose of microcarriers used, and the mode of placement of the magnet, were similar to those described above. Mice were killed 1 h after the injection of the microcarriers. The internal organs harvested included heart, liver, lung, kidney, spleen, and femur muscles.

##### Histological Studies.

Specimens were treated with 4% paraformaldehyde, dehydrated through a graded series of ethanol, washed with xylene, and embedded in paraffin wax. Serial 10-µm slices were made. Microcarriers containing iron were determined in histological samples by the Perls histochemical method. Paraffin was removed from the slices, and the slices were treated with hydrochloric acid alcohol for 30 min. After that, a solution of ferrocyanide was added and the samples were incubated for 10 min and then rinsed in distilled water. Background staining was done with hematoxylin and eosin. Morphologic analysis of the histological samples was done with an Olympus digital image analysis system.

##### Magnetometry

Pieces of organs were weighed, placed in a special vial, and dried in a vacuum oven at 20 °C for 4 h. Their magnetization was measured with an EZ11 vibration magnetometer (Microsense Inc., Lowell, Massachusetts, USA). The magnetite concentration in the samples was calculated from a preconstructed calibration plot.

##### In Vivo Biocompatibility

In the major sets of experiments, we evaluated the effects of intravenous carrier administration on the animals’ hematological parameters and biochemical serum markers. Male and female mice weighing 20 g were injected intravenously with the following:

(1) MNPs (BSA-TA) carriers diluted in saline solution (*n* = 10),
(2) (BSA-TA) carriers diluted in saline solution (*n* = 10)
(3) Saline (0.9% NaCl) as the control (*n* = 10).

##### Heart and Lung Function Study.

The heart rate, electrocardiography, breathing frequency, and SpO_2_ were measured in all groups immediately before injection, after 5–10 min, and after 24 h with an MP150 data acquisition and analysis system (BIOPAC Systems, CA, USA). Arterial blood pressure was measured with a CODA noninvasive blood pressure analyzer (Kent Scientific Inc, Torrington, CT, USA).

##### Hematological and Biochemical Parameters.

Blood was drawn for hematology analysis by a standard saphenous vein blood collection technique. A PCE-90Vet hematological autoanalyzer (HTI Inc., Powell, OH, USA) was used to determine hematological parameters such as hemoglobin (HGB), red blood cells (RBC), mean corpuscular volume (MCV), mean corpuscular hemoglobin (MCH), hematocrit (HTC), mean corpuscular hemoglobin concentration (MCHC), white blood cells (WBC), and platelets (PLT). Whole blood was centrifuged twice at 3000 rpm for 10 min to separate serum. Serum biochemical analysis was carried out with a DRI-CHEM 4000ie biochemical analyzer (Fuji Inc., Tokyo, Japan) to determine the levels of total bilirubin (TBIL), alkaline phosphatase (ALP), and alanine transaminase (ALT) in order to evaluate liver function.

##### Complement Study

The complement system was studied by enzyme-linked immunosorbent assay (ELISA) by using a commercial ELISA kit (TCC, Human, ELISA kit; Hycult Biotech, Netherlands). The study used blood plasma from four healthy donors. Plasma samples were obtained by centrifugation (3000 rpm for 15 min at room temperature) with an anticoagulant (sodium citrate). Microcarriers were added to plasma samples at two concentrations (1 × 10^9^/mL and 2 × 10^9^/mL), and the samples were incubated at 37 °C for 2 h with gentle stirring.

## 3. Results and Discussion

### 3.1. Preparation and Characterization of Nanocomposite Carriers

Controlled biodistribution of carriers in the organs by using magnetic field gradients was ensured by using MNPs in the preparation of the carriers. The average size 11 ± 3 nm were determined using DLS method (Figure S1a,b).

Vasir et al. showed that effective delivery of magnetic nanoparticles via systemic administration requires such a magnetic force as to compensate for the force of linear blood-flow rates of ~0.05 cm/s in the capillaries to 10 cm/s in the arteries and 50 cm/s in the aorta. To overcome this difficulty, magnetic nanoparticles with higher magnetic moments or magnets that can provide higher magnetic field gradients are required [30]. For increasing the loading of MNPs, FIL was used which consisted of the successive cyclic loading of MNPs and Cy7-conjugated BSA into vaterite particles. [31]. Figure 1 shows the obtainment of particles loaded with magnetite and with Cy7–BSA.

FIL allows the adsorption of a twofold larger amount of magnetite (13%) to occur, as compared to the adsorption (4%) and coprecipitation (3%) methods [31]. After receiving the particles, a (BSA-TA-(BSA–Cy7-TA)_2_/BSA-TA) shell was formed on their surface (Figure 1). After the particles were prepared, (BSA-TA-(BSA–Cy7-TA)_2_/BSA-TA) shells were formed on their surface (Figure 1). As the BSA-TA shell forms during macromolecule complexation through hydrogen bonds without the use of polyelectrolytes, the inclusion of negatively charged magnetite particles in the shell [32] is not effective. As a consequence, the only way to incorporate particles into the carrier is by inserting them into the core, which is another argument for the use of FIL to load the particles. The carriers were visualized with a fluorescence tomograph by using Cy7-conjugated BSA. The characteristics of the resulting structure are shown in Figure 1.

The 400–600 nm carriers that had formed on the vaterite particles with a large number of MNPs did not collapse during drying (Figure 1b,c). This indirectly indicates that the bulk of the magnetite particles was distributed on the surface of the vaterite particles. A shell/crust of magnetite was formed, which retained its shape after the dissolution of the core. These data have a good agreement with previously obtained result [31]. In comparison with the (BSA-TA) carriers (Figure S2), they do not retain their shape when the water dries.

Despite the inclusion of a sufficient amount (0.21 mg) of Cy7, the fluorescence spectra of the initial samples show that the carriers were nonfluorescent. Perhaps this was due to the “quenching” effect arising from the binding of the dye to the magnetite particles, as well as to the BSA-TA shell itself [33]. TA is a quenching molecule for BSA [30]. Previous studies have rarely used MNPs as fluorescence quenchers. Citrate-capped Fe_3_O_4_ NPs interact with doxorubicin through electrostatic attraction, thereby quenching their fluorescence. To confirm this theory, the fluorescence intensities of the MNPs (BSA–Cy7/TA) and (BSA–Cy7-TA) carriers, BSA–Cy7, and pure Cy7 (as controls), were compared after incubation for 0.1, 0.5, 1, 2, 5, 16, 24 and 40 h in physiological saline, blood, and plasma. The carriers were sufficiently stable in physiological saline, and the dye was firmly bound in the shell (Figure 1d). However, when the samples were incubated with plasma and blood, the fluorescence of the samples increased gradually, peaking at 24 h. Because this was not observed for the carriers in physiological saline, the effect can be explained by the gradual destruction of the carriers by plasma enzymes and cells (in the case of blood), and, as a result, by the destruction of the bound MNPs (BSA–Cy7/TA) complex, which led to fluorescence quenching (Figures S3 and S4). The results show that both the high magnetite concentration and the shell led to fluorescence quenching, which was not observed when either was absent. Thus, the obtained carriers can serve as indicators of their destruction in the body.

To define biocompatibility, BSA-TA and MNPs(BSA-TA) carriers were used for viability study for two cell lines (Figure S5): cancer cell Hela and fibroblasts. The BSA-TA and MNPs(BSA-TA) carriers were added to cells at concentrations of 10 and 50 carriers per cell. The analysis of data allows us to conclude that the increasing number of carriers per cell was led to decreasing viability of both types of carriers. But it is necessary to remark that all carriers did not significantly affect cell viability, and there were not remarkable changes of viability between different types of carriers. Therefore, the carriers are acceptable for in vivo application.

### 3.2. Organ Distribution

A number of “biological barriers” protect the body against foreign materials, including injected therapeutic and contrast agents, keeping carriers from reaching their intended destinations [34]. These barriers can restrict the function of carriers by blocking their movement, causing physical changes to them, or by inducing a negative host response by using biochemical signaling [35]. Upon intravascular administration, carriers immediately encounter blood, a high-ionic-strength, heterogenous solution that can induce carrier agglomeration, altering their magnetic properties and inducing particle sequestration. Additionally, carriers can nonspecifically interact with plasma proteins (which can trigger the adaptive immune system), extracellular matrices, and nontargeted cell surfaces while in the bloodstream [36]. In each case, the carriers are in danger of prematurely binding to or being taken up by cells before reaching its target tissue. In addition to coping with the vascular environment, carriers must overcome various anatomical size restrictions, which limit carrier access to target tissue (e.g., extravasation of lymph-targeting carriers from the blood vessels) [34]. The organ distribution of carriers depends on their size and biochemical and physical properties [37].

**Figure 2:**
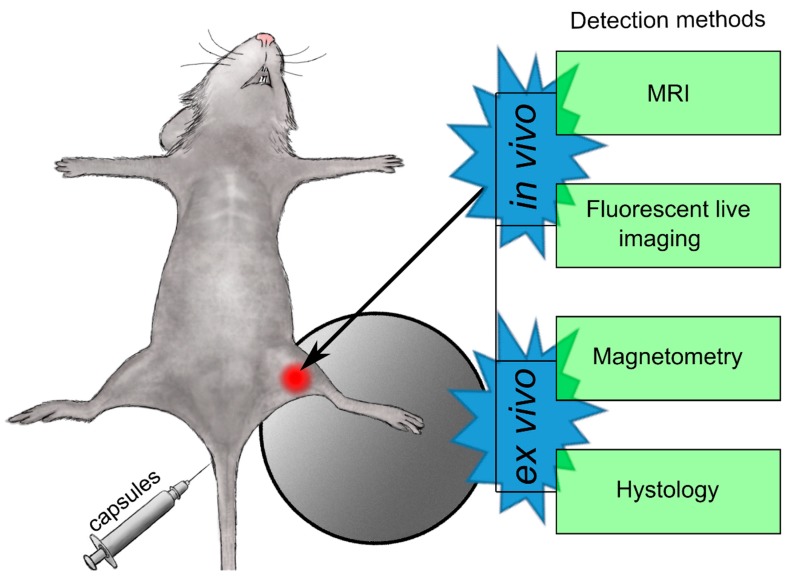
Schematic illustration of in vivo administration of the carriers and subsequent analysis using combined imaging instruments, magnetometry, and histology to elucidate carrier biodistribution.

As a result, the biodistribution of the submicron carriers obtained with a new biodegradable shell consisting of BSA and TA under conditions of an applied external magnetic field to one of the animal’s haunch (left) was investigated. The delivery efficiency to the haunch with a magnetic field gradient was evaluated using in vivo (MRI, fluorescence live imaging) and ex vivo (magnetometry and histology) methods (Figure 2).

#### 3.2.1. MRI

**Figure 3:**
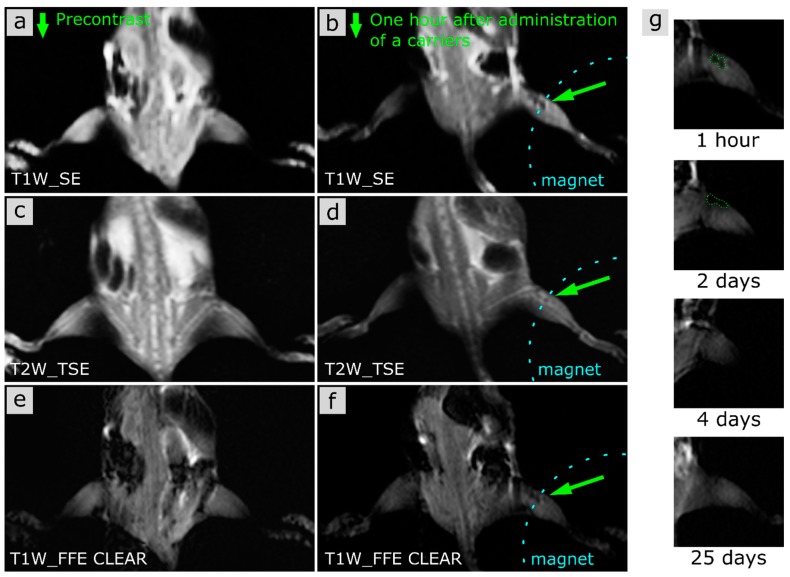
MR T1 and T2 images of the mouse. (**b**,**d**,**f**)—1 h after intravenous injection of a microcarrier suspension; (**a**,**c**,**e**)—control mouse without any injections. The dotted blue line shows the area of magnet application, (**g**)–T1W_FFE CLEAR images of paw 1 h, 2, 4, and 25 days after intravenous injection of a microcarrier suspension.

At the first stage of the study, evidence of “magnetically attracting” carriers to one of the paws was assessed using an MRI study. Submicron carrier suspension was introduced into the tail vein. The left paw was fixed on the magnet for 1 h and after that MRI was performed. The results are shown in Figure 3. In the region of the muscle that is located above the edge of the magnet (dotted blue line), the contrasting regions (the green arrow) are most likely to be carriers (Figure 3b,d,f). Two days after injection MR signal intensity (SI) from dark spots in the haunch (left) increased, that means contrast is decreased. After 4 days, the contrast of the region of interest was very low and could be detected using only T2 FFE pulse sequence, which is quite sensitive to the presence of MNPs.

The MR contrast was observed also in the liver (Figure S6). The MR signal from liver was decreased for 2 days and then increased on the fourth day. This suggested that a lot of magnetite distributed in the liver. The MR signal from liver received a normal value for 25 days after injection. These data have a good correlation with previously published results [38].

#### 3.2.2. In Vivo Fluorescence Lifetime Imaging

Using an IVIS live imaging system, we next examined carrier biodistribution under the influence of an external magnetic field applied to a mouse paw.

**Figure 4:**
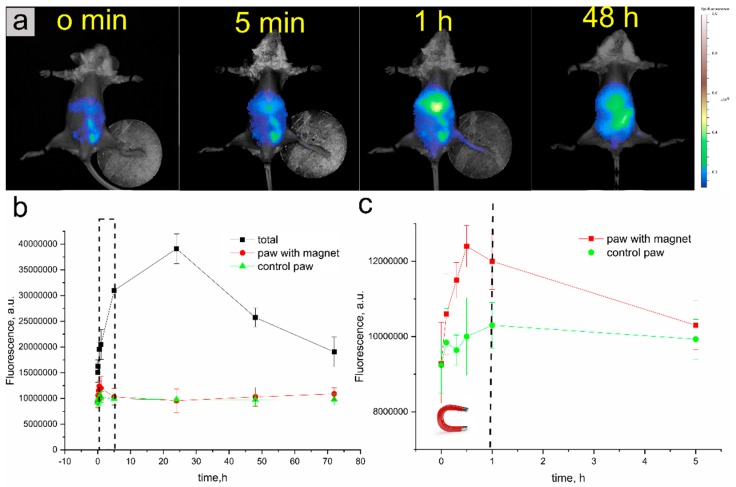
The organ distribution of carriers under the effect of a magnetic field applied to a mouse paw (real-time visualization). (**a**), whole-body fluorescence imaging and distribution of carriers before and after injection into the mouse tail vein. (**b**), change in the total fluorescence signal of the mouse and signals received from both paws within 72 h. (**c**), change in the fluorescence signal from the moment of carrier administration to 5 h (area marked with a dotted red line in Figure 4b).

The organ distribution of MNP_S_(BSA-Cy7-TA) carriers after administration at different time intervals (Figure 4a) (0 min [control, before carrier administration], immediately after administration [5 min], 1 h, and 48 h). The relative fluorescence intensity was calculated with account taken of the initial amount of fluorescence intensity for the mice before the carrier was inserted. The magnet was removed at 60 min after carrier administration (dotted black line in Figure 4c).

The carriers were nonfluorescent at the time of administration (Figure 4). Since the introduction of the carrier, the signal in the left paw with the magnet increased and was higher than in the intact right paw until the magnet was removed. The level of fluorescence in the paw with the magnet was increasing from the moment of administration, and at the point of 0.5–1 h, it was already possible to see a noticeable signal in the images of the whole body (Figure 4a), which was most likely due to the gradual release of the dye and the natural destruction of the carriers. The fluorescence intensity in the intact right paw increased only slightly after injection.

After the magnet was removed, the level of fluorescence in the paw to which a magnet had been applied started to decrease, approaching the level in the intact paw. This was due to the gradual elution of the particles from the vessels in the paw. Yet after 1 h the fluorescence level of the whole body of the mouse increased significantly, peaking after 24 h, which according to previous in vitro studies indicates the gradual destruction of the shell and the MNP–Cy7 complex. Thus, the results obtained in vivo mirror the in vitro data on the stability of carrier fluorescence in various biological fluids (Figure 1d). The strongest intensity came from the liver area after 24 h of intravenous injection (Figure 4a).

#### 3.2.3. Histological Studies and Magnetometry

**Figure 5:**
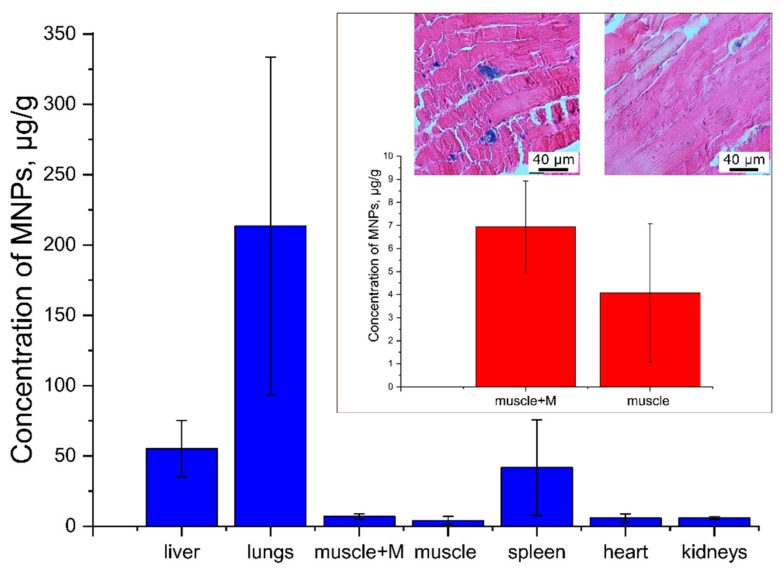
The magnetite tissue distribution measured by magnetometry. The predominant accumulation of magnetite in the lungs, liver and spleen. At the callout the concentration of magnetite in the intact muscle and the muscle exposed to the magnetic field as well as the histological picture of these muscles is presented (in insert). An increase in the concentration of magnetite by 70% compared with the intact limb was observed. Microaggregates of magnetite particles in the capillaries and venules were found.

For a better understanding of carrier distribution, mice were killed 1 h after the magnet had been removed and the MNP concentration in the collected organs was measured by magnetometry. Analysis of the tissue distribution of MNPs (BSA–Cy7/TA) shows that the carrier predominantly accumulated in the lung, liver, and spleen (Figure 5).

The accumulation of nano- and micro-particles in the liver and spleen is a well-known fact and has been described for many objects, such as polymer-coated microcarriers and liposomes [1,39,40]. The effect of size on carrier biodistribution is organ-specific and nonlinear [41]. This is due in part to the organ-specific physical and physiological barriers that systemically administered carriers encounter [42]. Studies on liposomes have shown that splenic sequestration of particles decreases linearly with decreasing of particle size [43,44]. For the liver, the dependence on size is also nonlinear. While larger carriers are sequestered in the liver (consistent with observations in the spleen), very small carriers (less than 70 nm) can pass through the sinusoidal fenestrations in the liver and be entrapped by the underlying parenchymal cells [11]. For polymer microcarriers obtained by LBL technology, significant accumulation in the liver and spleen has also been observed [10]. The absorption of nanoparticles by macrophages is usually considered the main mechanism of accumulation of foreign materials in the liver [41,42,45]. The tissue distribution of the magnetite-containing microcarriers is shown in Figure 5.

There is much less information in the literature about the accumulation of particles in the lungs. Such accumulation was described for silica-coated magnetic nanoparticles [43], titanium dioxide nanoparticles [44] and other similar structures [46,47]. Absorption by tissue macrophages is considered the main mechanism of particle accumulation in the lungs, similarly to what is observed in the liver [44,46]. However, in our case, the concentration of carriers in the lungs was very high (about four times higher than that in the liver and five times higher than that in the spleen). We believe that this phenomenon cannot be explained only by the absorption of particles by macrophages, because the main pool of phagocytes in the lungs is represented by alveolar macrophages with no direct contact with the blood.

The most probable cause for the intrapulmonary accumulation of particles is the features of their movement through the lung capillaries. It is known that the lungs function as a microfilter and retain most of the blood cell aggregates and other foreign objects [11]. Although the diameter of the carriers is smaller than that of the capillaries, even unstable aggregates consisting of two or three particles can embolize the pulmonary capillary. Furthermore, the distribution of particles in the bloodstream may be important. The blood flow velocity is maximal in the center and minimal or equal to zero at the periphery of the capillary [12]. Particles trapped in the parietal area of the bloodstream slow down their movement significantly or even completely stop.

This process can be reversible, and eventually, the concentration of “parietal” particles decreases. However, when the particles adhere to the endothelial cells more strongly, they may accumulate long-term in the lungs. If the particles activate platelets and/or plasma clotting factors, adhesion to the endothelium may lead to capillary thrombosis with the subsequent organ dysfunction. In our case, there was no evidence of pulmonary capillary thrombosis, because no subsequent lung dysfunction was observed.

It should also be noted that when the particles accumulate in the capillaries through the “hemodynamics mechanism,” this process should be more pronounced in pulmonary tissue owing to the “first pass effect,” because 100% of the substance administered intravenously passes through the lung capillaries.

Our analysis of the accumulation of carriers in the kidneys, heart, and intestines shows that their concentrations in these organs are proportional to the degree of their vascularization. This fact indicates the mainly intravascular character of carrier circulation, especially early after administration.

The concentration of the carriers in muscle tissue is to be analyzed separately, because in this study, the hip muscle was the point of targeting of magnetically controlled objects. The results are shown in the inset of Figure 5. The choice of the hip muscle was primarily due to the anatomical availability of this area for the application of the magnetic field. Although the blood flow in muscles without physical activity is small, using this tissue has an undisputable advantage, i.e., the possibility of comparing the concentration of carriers in the intact muscle and in the muscle placed in a magnetic field gradient in the same animal at the same time.

One can see that the concentration of carriers in the muscles was lower, as compared to that in the other organs. However, in the paw placed in a magnetic field, the concentration of the particles was higher by 70% than it was in the intact paw. Probably, the mechanism of accumulation is based on the displacement of carriers by the magnetic field gradient from the axial region of the capillary to its periphery, where, as already mentioned, the blood flow velocity is lower.

The histological analysis of the paws with and without the magnet showed that the muscle fibers were located in parallel and that the transverse striation was retained. Throughout the thickness of the slice, in the muscle tissue of the paw to which the magnet was attached, we observed diffusely located aggregates of magnetite (Figure 5, inset).

### 3.3. Effect of Carrier Administration on Functional and Biochemical Parameters

Because the in vivo toxicity of the BSA-TA carriers has not been previously described in the literature, this study was aimed at a comparative assessment of the in vivo toxicity of the BSA-TA and MNPs(BSA-TA) carriers in mice. The effects of intravenous infusions of the carriers on the hemodynamic variables were studied first. The effects of the intravenously administered carriers on the hematological parameters and biochemical serum markers were also examined.

#### 3.3.1. Hemocompatibility

A study of the effect of micro- and nanoparticles on the blood system (hemocompatibility) is obligatory in toxicological research, because all such objects are directly introduced into blood and have permanent contact with it. The complement and clotting systems are the most sensitive blood systems, which interact with the particles and can be activated by them.

The complement system (CS) is a complex of proteolytic enzymes that is activated by foreign proteins in blood. The system can be activated, separately or simultaneously, through three independent pathways: the classical, the alternative, and the lectin.

Usually, the CS can be activated by bacteria, viruses, and tumorous or infected cells. However, CS activation can take place in response to any foreign object, including nanoparticles, synthetic materials, and biopolymers. The influence of the surface functional groups of nanomaterials on CS activation has been discussed. Biopolymers carrying surface hydroxyl groups or natural polysaccharides, such as cellulose or dextran, have been shown to activate the CS in vitro and in vivo [48,49]. The CS can also be activated also by gold [50], and ferromagnetic nanoparticles [51], carbon particles, micelles and liposomes, etc [50].

**Figure 6:**
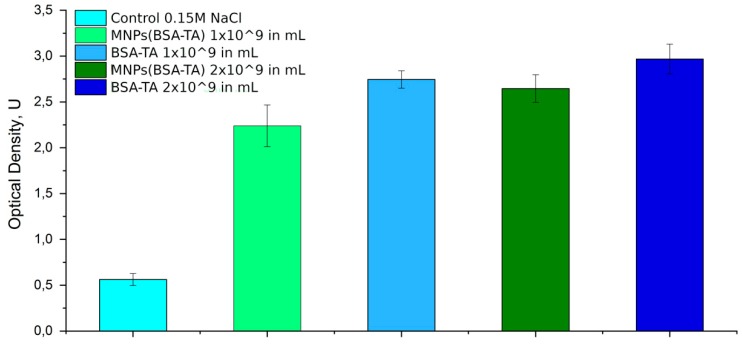
The effect of BSA-TA and MNPs(BSA-TA) microcarriers on complement system. Determination of membrane attacking complex (MAC) concentration in human serum with ELISA assay. Dose-independent activation of complement system with both types of carriers. Levels of MAC in all experimental groups are significantly higher compared with the control group.

In the case of intravenous introduction of nanomaterials, the main pathway of CS activation is the alternative one or (less often) the lectin one. The classical pathway is not launched, because it requires the presence of specific antibodies. All the three pathways converge at the stage of C3-convertase generation, after which a SC5b-9 complex is synthesized. This bioactive agent, called the membrane attack complex (MAC) or the terminal complement complex (TCC), can be used as a marker of CS activation. In addition to SC5b-9, other substances such as C3a or C5b can also be used for this purpose. In this study, we are focused on measuring the overall level of complement activation, by using SC5b-9 as the main marker of activation of all pathways. The results of these studies are shown in Figure 6.

One can see that both types of carriers led to an increase in the SC5b-9 level. It should be noted that the activity of SC5b-9 was significantly higher than that in the control for both concentrations of microcarriers (1 × 10^9^/mL and 2 × 10^9^/mL). This activity increase was similar for both doses of microcarriers, which points to a dose-independent character of the effect.

In our case, the most likely mechanism of CS activation by the carriers is the alternative pathway. It starts with a hydrolysis of the thioether bond of the C3 component, which can be caused by the particle directly or by the plasma proteins adsorbed on its surface [52]. It is possible that the adsorbed protein, forming the specific “protein corona,” plays a decisive role in the biological effects of the particle [53,54].

The result of the hydrolysis of C3 is the formation of the C3a component, which is released into the plasma, and of the C3b component, which is adsorbed on the carrier’s surface and accelerates the further reaction of assembling C5b-9 in plasma. It should be noted that CS activation may have different physiological consequences. The activated components of the complement (especially anaphylatoxins C3a and C5a) have high biological activity and cause histamine release, leukocyte chemotaxis, an increase in vascular permeability, and a contraction of smooth muscles. They also cause symptoms similar to anaphylaxis, which can lead either to mild vascular reactions or to severe respiratory and circulatory disorders [55] Neun BW2018. Along with CS activation, the cross-activation of the kallikrein–kinin system, platelets, and clotting is possible, which is fraught with uncontrolled disturbance of hemostasis and hemodynamics [55]. However, under physiological conditions, there are effective and rapid mechanisms for controlling the activity of anaphylatoxins, as well as inhibitors of C1 and C3-convertases [56]. Therefore, the resulting biological effect is determined by the balance between the degree of activation and the level of inhibition factors.

We note that activation of the complement system by nanoparticles occurs quite often and is not a factor that makes clinical use of these particles impossible. So, it is known that a number of drugs approved by the FDA, causes activation of complement, for example, liposomal anticancer drugs («Doxil», «DaunoXome», «Taxol» (Paclitaxel), «Taxotere» (Docetaxel) [57,58,59], «Ambisome» [60], most of the iron containing drugs for intravenous administration [61,62], etc. For some of them, anaphylactoid and system inflammatory reactions are described [59,60,61,62]. However, in general, the potential benefits of using these particles exceed the harm from this side effect. In our case, the carriers administration did not lead to serious hemodynamic reactions and activations of systemic inflammation (see 3.6. and 3.11.), so we can assume that activation of complement is not an absolute obstacle to further study and clinical use of the synthesized carriers.

#### 3.3.2. Hemostasis

The clotting of blood is activated during the interaction of the coagulation system with foreign surfaces (internal pathway) and with specific tissue activators, which represent a complex of apoprotein III and phospholipids (external pathway). Various nano- and micro-particles can activate the clotting cascade both in the internal and in the external pathway [63]. Intravascular coagulation causes thrombosis and partly or completely blocks blood vessels, leading to tissue [29,64].

However, there may be an opposite reaction to the introduction of nanoobjects. They can have an anticoagulant effect and provoke bleeding [65,66,67]. Most widespread tests to assess hemostasis are partially activated thromboplastin time (PATT) and prothrombin time (PT). It was shown [48] that these in vitro clotting tests are highly predictable in vivo. The absence of influence of nanoobjects on the coagulation system indicates that they are biocompatible and can be used in vivo.

**Figure 7:**
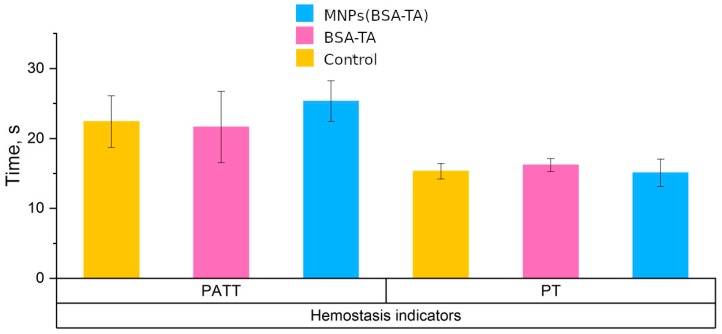
Effect of microcarriers on hemostasis: partially activated thromboplastin time (PATT) and prothrombin time (PT) in human blood plasma. Dose of carriers is 1 × 10^9^/mL. No effect of carriers on PATT and PT is present.

The results of the hemostasis study are presented in Figure 7.

We can see that neither PATT nor PT changed significantly when either type of microcarrier was added to the plasma. This indicates that these microcarriers do not affect clotting and are hemocompatible.

#### 3.3.3. Hematologic Study

The hematological toxicity of nanoparticles has been investigated intensely in recent years [68]. Studies have shown that there are various manifestations of hematological toxicity in particles. These include an increase or decrease in the number of blood cells (erythrocytes, leukocytes, and platelets), a change in the hemoglobin content, hemolysis, endothelial dysfunction, and activation or inhibition of the inflammatory response.

For instance, decreases in the number of red blood cells and in the content of hemoglobin may indicate bleeding or intravascular hemolysis [66]. In contrast, an increase in the number of erythrocytes indicates tissue oxygen deprivation or immune response activation [49]. An increase in leukocytes indicates is evidence of an inflammatory process. A decreased number of platelets may be a symptom of intravascular thrombosis [69].

**Figure 8:**
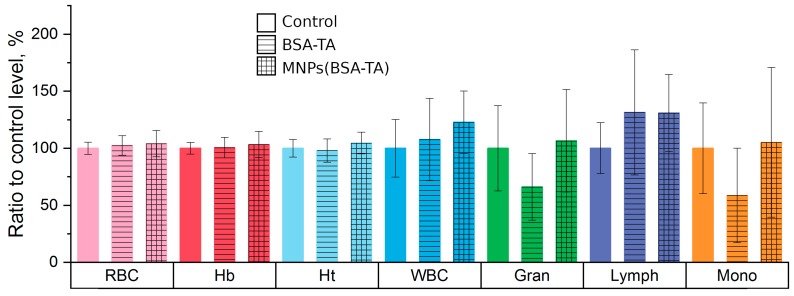
Effect of microcarriers on hematological parameters (data are presented as per cent of control group level). RBC—red blood cells, Hb—hemoglobin, Ht—hematocrit, WBC—white blood cells, Gran—granulocytes, Lymph—lymphocytes, Mono—monocytes. Both types of carriers did not affect levels of hemoglobin, red and white blood cells.

The results of the hematologic study are shown in Figure 8.

We did not find any significant changes in the hematological parameters with either type of microcarriers. This result indicates that the carriers are not hematotoxic at the doses used and are safe for the blood cells.

#### 3.3.4. Liver and Kidney Function Tests

In studies of the biological effects of nanomaterials, much attention has been given to the examination of the liver and kidney. This is because most of the intravenously injected nano- and micro-particles accumulate in the organs of the reticuloendothelial system such as the liver and spleen. Some types of particles can be highly concentrated in the kidneys also, but this pathway of excretion is not typical of nanostructures. Particle concentrations in the liver, kidneys, and spleen may exceed the systemic one by three to eight times, depending on the type of the particle. According to some authors, the accumulation of particles in the liver does not lead to its injury if the particles are made of biocompatible and biodegradable materials [51]. On the contrary, other researchers concluded that nanoparticles are foreign objects regardless of the materials used to make them and can damage the organs in which they accumulate. Also, they can form granulomas at the site of injection, especially in the liver, spleen, and kidneys. Subsequently, these changes are manifested as shifts in the biochemical parameters of blood [50].

**Figure 9:**
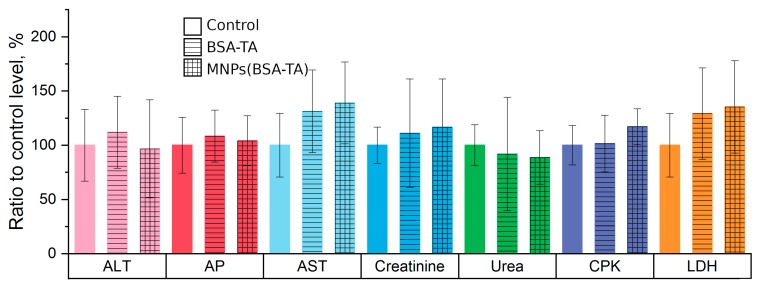
Effect of microcarriers on the level of biochemical markers (variables examined are shown as per cent of control group level). ALT—alanine transaminase, AP—alkaline phosphatase, AST—asparagine transaminase, CPK—creatine phosphokinase, LDH—lactate dehydrogenase. A statistically significant increase of LDH level and a tendency towards AST elevation was observed. Discussion in the text.

We used a common set of biochemical markers to assess the state of the liver and kidneys: urea and creatinine for evaluation of renal dysfunction [70]; and aspartic transferase (AsT), alanine transferase (AlT), and alkaline phosphatase (APF) for assessment of hepatic dysfunction [71]. However, it should be noted that the AsT level is not a strictly specific marker for liver disease and an increase in AsT can also be observed when organs other than the liver are damaged (e.g., myocardium) [72]. The effect of the microcarriers on the levels of the above markers is shown in Figure 9.

The changes in the concentration of hepatic damage markers in the group of animals treated with BSA–TA microcarriers were not statistically significant, although there was a noticeable tendency for increased AST. A similar tendency was observed in the animals receiving MNPs (BSA–TA) carriers. There was no increase in ALT or IV, but the concentration of ACT increased (in this group, the increase was statistically significant). We believe that the administration of carriers at the doses used does not damage the liver, whereas the isolated increase in AcT in the MNPs (BSA–TA) group may be a manifestation of myocardial damage. The latter was confirmed with other markers, as discussed below.

There were no changes in the concentration of these markers with either type of microcarrier.

#### 3.3.5. Lung Function Tests

The effect of various particles on the respiratory system is often increased when those particles are administered into the trachea in aerosol or suspension form [73]. At the same time, the study of the pulmonary function is also very important at intravenous administration of particles, because all foreign objects introduced intravenously pass through the lung capillaries, which filter and trap all adulterants. In addition, the particles themselves can be trapped in the lung blood vessels. In this regard, the diameter of the particles and their concentration in the suspension are very important, as is the presence of aggregates. Larger particles can block capillaries and cause ischemic damage to the lungs.

An analysis of the pulmonary function and saturation was carried out. We measured the respiratory system parameters immediately (after 5 min) and a day after the administration of the microcarriers. The choice of these time points allowed us to track both early and late disease. Early disease can be caused by pulmonary capillary embolism and acute vascular reactions to the injected object, and late disease may be associated with the activation of local and systemic inflammation.

**Figure 10:**
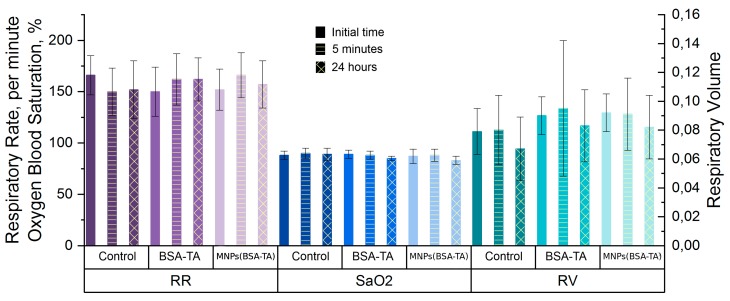
The effect of microcarriers on the respiratory system function. RR—respiratory rate, SaO2—oxygen blood saturation, RV—respiratory volume. No effect of the carriers on respiratory system function was observed.

In this study, we have not found any significant changes in the respiratory system (Figure 10).

The parameters of external respiration (RR and TV) and blood oxygenation obtained at 5 min and 1 day after the administration of both types of carriers did not differ from those recorded before administration. The absence of an injuring effect on the lungs is probably related to the small size of the carriers and the small number of aggregates in the suspension, which allows microcarriers to pass through the pulmonary capillary filter.

#### 3.3.6. Heart Function Tests

Much attention is now paid to the cardiovascular effects of nano- and micro-objects. Nano- and micro-particles accumulate in the cardiac muscle in smaller amounts than they do in the liver and spleen, but their cardiovascular effects can be implemented through various mediator systems and biochemical processes. The main mechanism of toxicity of iron-containing particles is the activation of lipid peroxidation, leading to membrane damage and failure of membrane-bound enzymes [74,75,76]. In addition, the cardiotoxic effects may be caused by nonspecific activation of the immune response [75]. As already mentioned, nanoparticles can activate the inflammatory response directly (causing the release of cytokines from the immunocytes) or indirectly through the complement and the kallikrein-kinin systems [55,77]. The activation of inflammation may cause different cardiovascular responses [55] such as tachycardia, arrhythmias, arterial hypotension, and heart failure.

**Figure 11:**
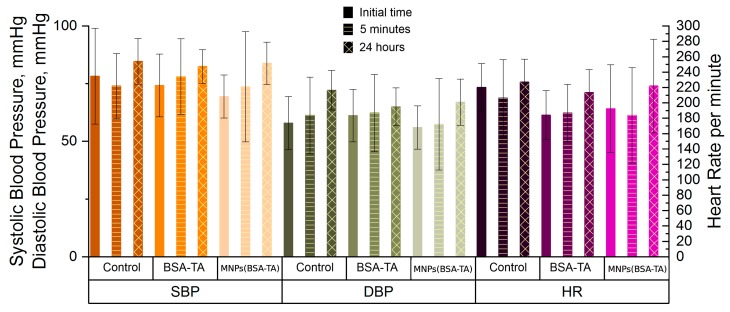
Effect of microcarriers on cardiovascular system. SBP – systolic blood pressure, DBP – diastolic blood pressure, HR – heart rate. An increase in SBP and DBP in the group receiving MNPs (BSA-TA) carriers and DBP in the control group was observed. Discussion in the text.

The results of the study of the cardiovascular system responses after microcarrier administration are shown in Figure 11.

In all groups, there was a tendency for an increased heart rate at the point “24 h”; however, this effect was not statistically significant. At this point, we observed an increase in systolic and diastolic blood pressure in the group receiving MNPs(BSA–TA) and an increase in diastolic blood pressure in the control group. We believe that the increase in blood pressure is not a specific effect of the carriers but the result of the stress underwent by the animals the day before. This assumption was confirmed by the fact that similar changes were noted both in the experimental group and in the control group and by the tendency for an increased heart rate in all groups at the point “24 h.”

Heart damage markers have also been determined to assess the effects of the microcarriers on the cardiovascular system. We can observe a moderate but statistically significant increase in the level of aspartate aminotransferase (AsT) and lactate dehydrogenase (LDH), as well as a tendency for an increased creatine phosphokinase (CPK) level in the group receiving MNPs(BSA–TA). Furthermore, a tendency for increased levels of AsT and LDH was detected in the animals receiving BSA–TA carriers, but it was not confirmed statistically. This results may indicate subclinical myocardial damage, not manifested as violations of the hemodynamics or heart rhythm. Myocardial damage may be due to the above-mentioned activation of LPO and to immune inflammation. A combination of these factors is possible, but the induction of free-radical processes mediated by iron seems to play a more important role, as compared to the activation of nonspecific immune responses. This is evident from the fact that there were no significant symptoms of systemic inflammation such as leukocytosis, tachycardia, and/or arterial hypotension.

It should be noted that the myocardial damage unrelated to injury of other organs cannot be explained only by the activation of systemic processes such as LPO or inflammation. In those cases, usually most of the inner organs are injured, but in our investigation, no significant damage was found. Neither can cardiac damage be related to the accumulation of the carriers in the myocardium, because the carriers accumulate in other organs (such as lung, liver, and spleen, according to the data in Figure 5) in much larger quantities than in the myocardium but do not cause their dysfunction. Probably, the peculiarities of myocardial metabolism are important. One of these peculiarities may be the higher concentration of fatty acids in the myocardial cells. It is known that these substances are important for the energetic metabolism of the cardiac muscle. Various toxic products (e.g., dicarboxylic acids) damaging membranes and subcellular structures can be formed from fatty acids under conditions of iron-activated oxidative stress.

In addition, a possible reason for cardiotoxicity can be capillary embolization. Despite the small size and stability of the carriers in vitro, embolization may be caused by the partial aggregation of carriers and by the increase in their hydrodynamic radius during interaction with plasma proteins [78]. This process may be clinically insignificant for hypoxia-resistant organs such as liver or muscle, or for tissues with a well-developed vascular network (lungs), but it can be more critical for the myocardium. An indirect confirmation of this possibility is the tendency toward an increase in the level of the myocardial damage markers in the group that received magnetite-free carriers.

To conclude the discussion of the changes in the myocardium, we note again that the increase in the markers of myocardial damage was insignificant and did not disrupt the function of the heart. However, these changes may limit the further increase in the dose of the carriers.

## 4. Conclusions

Our study shows the feasibility of preparing stable submicron-sized carriers with the low-cost biodegradable BSA and TA components, bonded by hydrogen interactions. The possibility of particle navigation under the action of an external magnetic field gradient was demonstrated with carriers containing high concentrations of MNPs (causing a high magnetic moment) and the Cy7 dye inside the core. The carriers were stable in salts but were gradually destroyed in plasma and blood. Organ distribution studies of the carriers under the conditions of an applied external magnetic field to one of the animal’s haunch (left) demonstrated that the carriers accumulated mainly in the lungs. It was also observed that in a paw placed in a magnetic field, the concentration of the carriers was higher by 70% than in non-addressed paw. The current study suggests the flexible and nontoxic nature of BSA-TA carriers. Intravenous administration of MNPs(BSA-TA) carriers on hematological parameters, the complement system, hemostasis, respiratory and cardiovascular systems, kidney and liver function were found to have no adverse effect. Thus, the investigated carriers are demonstrated to be hemocompatible. They do not affect blood coagulation and, in given doses, are safe for red blood cells. They activate the complement system, but do not cause a significant systemic inflammatory response, as evidenced by the absence of changes in the level of white blood cells. Finally, no significant changes were found in the respiratory system, kidney and liver functions, and the increase in myocardial damage was not so significant.

## Supplementary Materials

The following are available online at https://www.mdpi.com/2073-4360/11/6/1082/s1, Figure S1. DLS of magnetite particles, Figure S2. SEM images of BSA/TA capsules (without MNPs), Figure S3. The effect of various biological fluids on the carrier stability, Figure S4. The effect of various biological fluids on the MNPs (BSA/TA) carrier stability after centrifugation, Figure S5. The percent of viable cells (a- Hela, b- fibroblasts) after incubation (free cells set as 100%), with added BSA-TA and MNPs(BSA-TA) carriers measured by Alamar blue method. The carriers were added to cells at concentration from 10 to 50 capsules per cell, Figure S6. MR T2 images of mouse before and 1 h, 2, 4, 25 days after intravenous injection of a microcapsule suspension. The dotted orange line shows the area of the liver.
