# Supplementary material for: Submicron-Sized Nanocomposite Magnetic-Sensitive Carriers: Controllable Organ Distribution and Biological Effects
Source: Polymers (Basel). 2019 Jun 25;11(6):1082. doi: 10.3390/polym11061082 (PMC6630964; doi:10.3390/polym11061082)
Supplement: Supplementary file 1 [file polymers-11-01082-s001.pdf]

Supporting Information

Submicron-sized nanocomposite Magnetic-sensitive Carriers: controllable Organ Distribution and Biological Effects

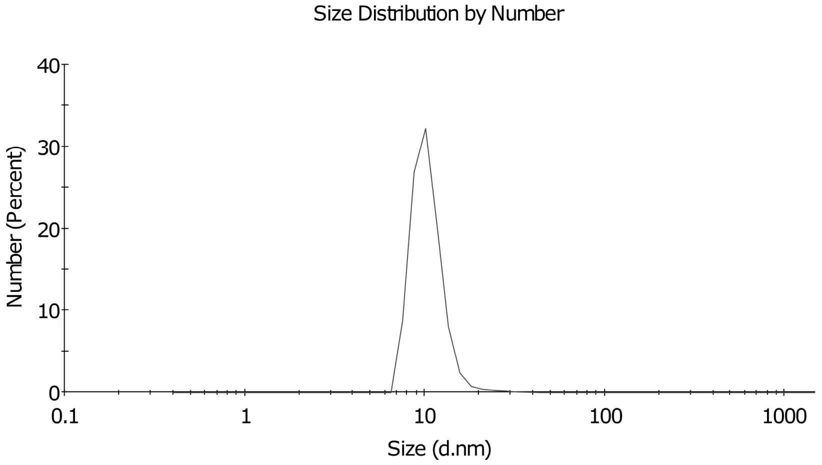

Figure S1 –DLS of magnetite particles

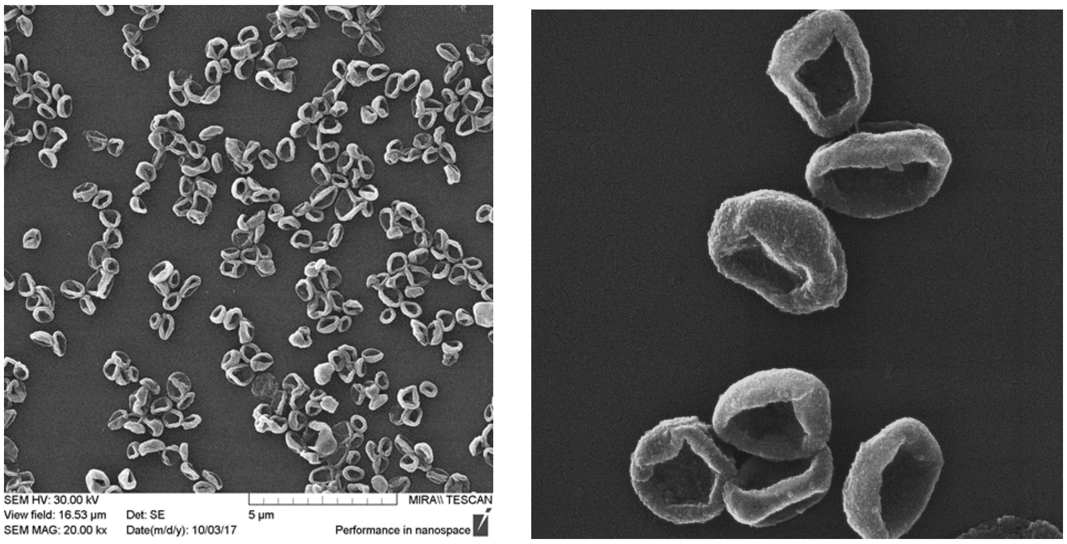

Figure S2 – SEM images of BSA/TA capsules (without MNPs)

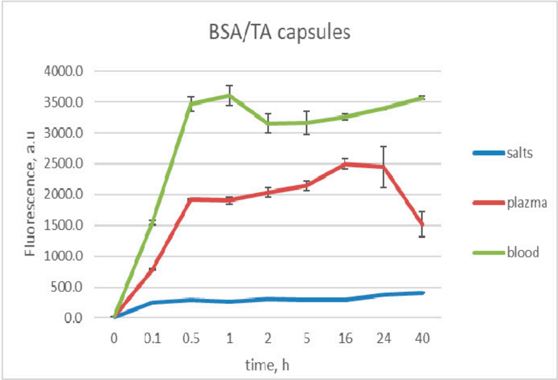

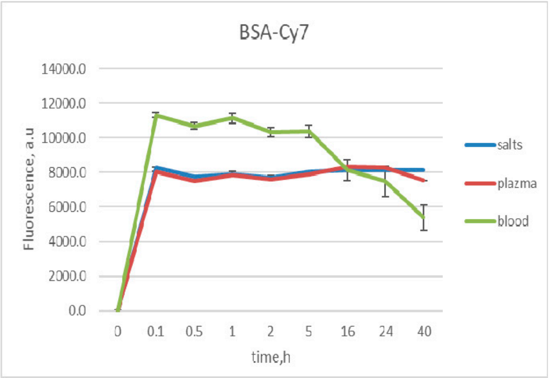

a **b**

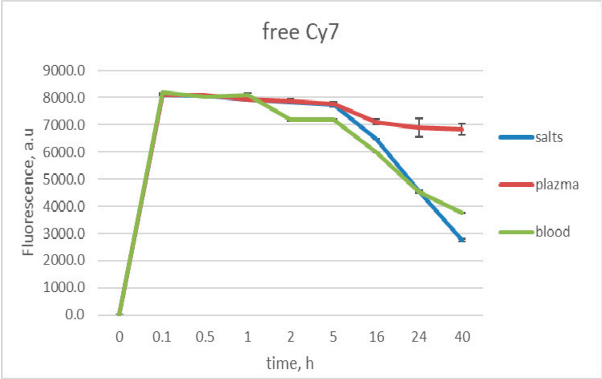

c

Figure S3 - The effect of various biological fluids on the carrier stability

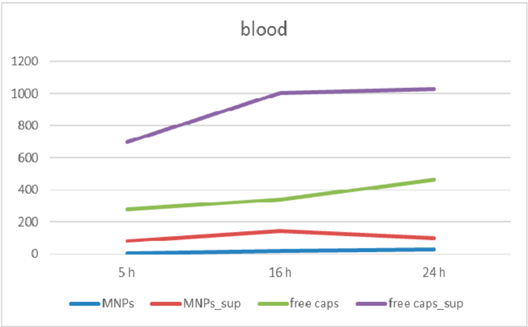

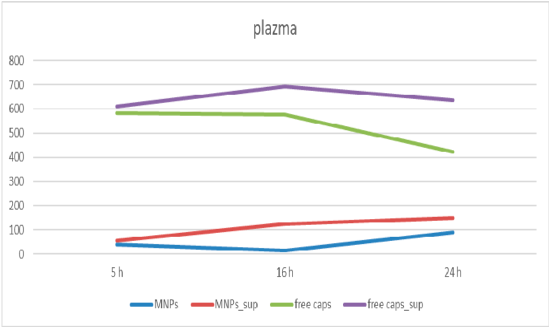

a **b**

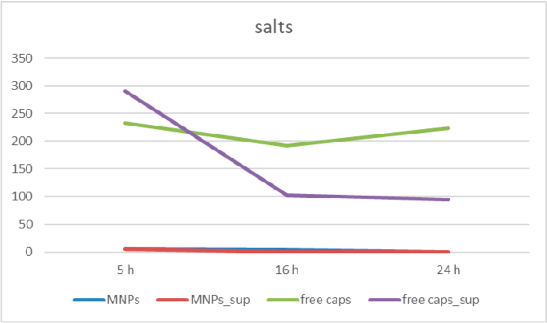

c

Figure S4 - The effect of various biological fluids on the MNPs (BSA/TA) carrier stability after centrifugation

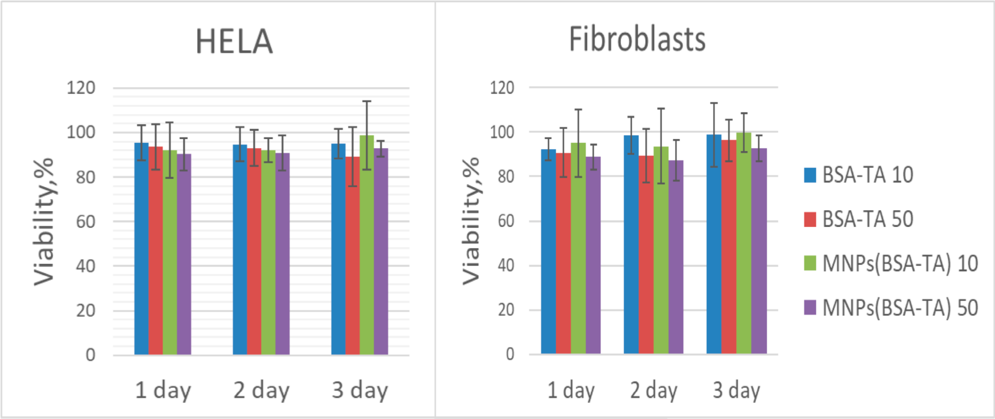

Figure S5 - The percent of viable cells (a- Hela, b- fibroblasts) after incubation (free cells set as 100%), with added BSA-TA and MNPs(BSA-TA) carriers measured by Alamar blue method. The carriers were added to cells at concentration from 10 to 50 capsules per cell.

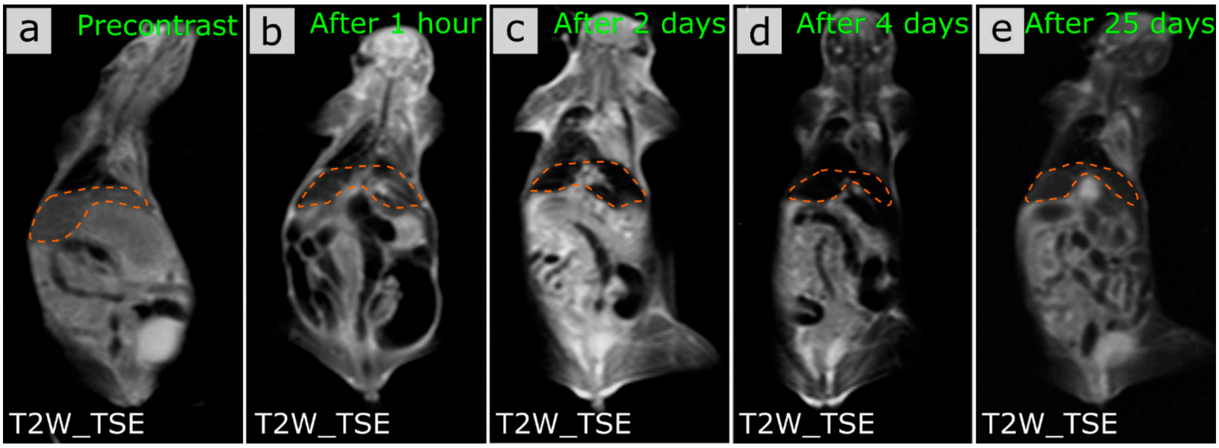

Figure S6 - MR T2 images of mouse before and 1 hour, 2, 4, 25 days after intravenous injection of a microcapsule suspension. The dotted orange line shows the area of the liver
